# Lifetime occupational exposure to metals and welding fumes, and risk of glioma: a 7-country population-based case–control study

**DOI:** 10.1186/s12940-017-0300-y

**Published:** 2017-08-25

**Authors:** Marie-Elise Parent, Michelle C. Turner, Jérôme Lavoué, Hugues Richard, Jordi Figuerola, Laurel Kincl, Lesley Richardson, Geza Benke, Maria Blettner, Sarah Fleming, Martine Hours, Daniel Krewski, David McLean, Siegal Sadetzki, Klaus Schlaefer, Brigitte Schlehofer, Joachim Schüz, Jack Siemiatycki, Martie van Tongeren, Elisabeth Cardis

**Affiliations:** 1 Epidemiology and Biostatistics Unit, INRS-Institut Armand-Frappier, Université du Québec, 531, Boul. Des Prairies, Laval, Quebec, H7V 1B7 Canada; 2 School of Public Health, University of Montreal, Montreal, Canada; 3 University of Montreal Hospital Research Centre (CRCHUM), Montreal, Canada; 4 Barcelona Institute for Global Health (ISGlobal), Barcelona, Spain; 5 Universitat Pompeu Fabra (UPF), Barcelona, Spain; 6 CIBER Epidemiología y Salud Pública (CIBERESP), Madrid, Spain; 7 McLaughlin Centre for Population Health Risk Assessment, University of Ottawa, Ottawa, Canada; 8 Oregon State University, Corvallis, Oregon, USA; 9 Monash University, Melbourne, Australia; 10 Institute of Medical Biostatistics, Epidemiology and Informatics, University Medical Center, Johannes-Gutenberg University Mainz, Mainz, Germany; 11 University of Leeds, Leeds, UK; 12 Unité Mixte de Recherche Epidémiologique Transport Travail Environnement Université Lyon 1/IFSTTAR, Université de Lyon, Lyon, France; 13 School of Epidemiology, Public Health and Disease Prevention, Faculty of Medicine, University of Ottawa, Ottawa, Canada; 14 Centre for Public Health Research, Massey University, Wellington, New Zealand; 15 The Cancer & Radiation Epidemiology Unit, The Gertner Institute, Chaim Sheba Medical Center, Ramat Gan, Israel; 16 Sackler Faculty of Medicine, Tel-Aviv University, Tel-Aviv, Israel; 17 German Cancer Research Center (DKFZ), Heidelberg, Germany; 18 International Agency for Research on Cancer (IARC), Section of Environment and Radiation, Lyon, France; 19 Institute of Occupational Medicine, Edinburgh, UK; 20 Centre for Occupational and Environmental Health, Centre for Epidemiology, University of Manchester, Manchester, UK

**Keywords:** Glioma, Occupational exposures, Metals, Welding fumes

## Abstract

**Background:**

Brain tumor etiology is poorly understood. Based on their ability to pass through the blood–brain barrier, it has been hypothesized that exposure to metals may increase the risk of brain cancer. Results from the few epidemiological studies on this issue are limited and inconsistent.

**Methods:**

We investigated the relationship between glioma risk and occupational exposure to five metals - lead, cadmium, nickel, chromium and iron- as well as to welding fumes, using data from the seven-country INTEROCC study. A total of 1800 incident glioma cases and 5160 controls aged 30–69 years were included in the analysis. Lifetime occupational exposure to the agents was assessed using the INTEROCC JEM, a modified version of the Finnish job exposure matrix FINJEM.

**Results:**

In general, cases had a slightly higher prevalence of exposure to the various metals and welding fumes than did controls, with the prevalence among ever exposed ranging between 1.7 and 2.2% for cadmium to 10.2 and 13.6% for iron among controls and cases, respectively. However, in multivariable logistic regression analyses, there was no association between ever exposure to any of the agents and risk of glioma with odds ratios (95% confidence intervals) ranging from 0.8 (0.7–1.0) for lead to 1.1 (0.7–1.6) for cadmium. Results were consistent across models considering cumulative exposure or duration, as well as in all sensitivity analyses conducted.

**Conclusions:**

Findings from this large-scale international study provide no evidence for an association between occupational exposure to any of the metals under scrutiny or welding fumes, and risk of glioma.

**Electronic supplementary material:**

The online version of this article (doi:10.1186/s12940-017-0300-y) contains supplementary material, which is available to authorized users.

## Background

The age-standardized incidence of primary malignant brain tumors is approximately 4.7–5.7 per 100,000 population worldwide, with rates reported to be higher in more economically developed countries [1, 2]. Twenty-four percent of brain tumors in adults are gliomas, with the incidence being slightly higher in men than in women. Glioblastomas and other malignant gliomas represent approximately 75% of malignant brain tumors [3]. For all ages and subtypes, the 5-year survival rate following diagnosis is between 20 and 30% in the U.S. [1]. Survival can vary greatly based on subtype, ranging from 3.3% for glioblastoma multiforme to over 70% for lower-grade gliomas. Aside from histologic subtype, the best predictor of survival is age at diagnosis, with older patients facing poorer outcomes.

The etiology of brain tumors is not well understood. Studies on possible risk factors for brain tumors have recently been reviewed [4]. Most studies show an increased risk of brain tumor after exposure to ionizing radiation. Based on positive associations between glioma and exposure to radiofrequency electromagnetic fields (RF-EMF) from wireless phones, RF-EMF have been classified as possibly carcinogenic [5]. Other risk factors that have been investigated include allergies and asthma, certain viruses, previous head injury, genetic factors, diet and smoking. As well, demographic factors such as race, sex and socio-economic status have been explored. To date, however, findings have been largely inconsistent [1–4, 6].

Workers in a number of occupations entailing exposure to neurotoxic and carcinogenic compounds have also been studied. Occupational exposure to metals has been of particular interest with respect to brain tumors owing to the ability of several metals to pass through the blood–brain barrier and to be up-taken via olfactory pathways into the brainx [7–9]. An increased risk of glioma has been reported in men with occupational exposures to arsenic, mercury and petroleum products [10]. Lead exposure has been linked to brain cancer mortality [11, 12] and glioma incidence [11–13]. In a cohort of Finnish women, elevated risks of brain-nervous system cancer were observed with occupational exposure to iron, chromium, lead and cadmium, albeit none of the risk estimates achieved statistical significance [14]. Brain cancer mortality was found to be elevated among chromium-exposed workers in Japan [15] and welders using chromium-nickel steel [16]. Glioma risk was associated with metal exposure as a group [17]. Other occupational studies document no association between glioma and exposure to metal [18] or lead [19, 20], or between brain cancer and lead [21]. Many of these studies were hampered by small numbers of brain cancer cases, crude occupational histories, or weak exposure information. A recent study reported an increased risk of brain cancer with environmental exposure to lead emissions from petrol [22].

Evaluating associations between occupational exposures and glioma is challenging. Occupational exposures are complex, and can include many different substances at varying concentrations. Therefore, detailed occupational histories and retrospective exposure estimates are required. Due to low incidence, large populations are needed to obtain a sufficient number of glioma cases for study. In earlier studies, this has led some researchers to group all brain tumors together instead of studying specific subtypes which may be linked to different risk factors. Given these issues, associations between glioma and occupational exposures are likely best studied in the context of a large case–control study with detailed occupational histories and exposure estimates.

In the late 1990’s, an international case–control study of mobile phone exposure and brain tumors was launched [23]. The INTERPHONE study included data from 13 countries. In addition to a detailed history of mobile phone use, data were collected on a number of other known and possible risk factors, along with detailed occupational histories. Seven of the 13 participating countries, comprising 10 study centers, constituted the INTEROCC study group established to evaluate the role of occupational exposures in brain tumors. Analyses focusing on exposure to solvents, combustion products, dusts and other chemical agents, and extremely low frequency magnetic fields (ELF) have been conducted [24–28]. Risks were close to null for all agents and glioma, other than for ELF which were associated with increased risk. Moreover, an analysis of occupational metal exposure and meningioma risk in INTEROCC [29], including data on 1906 adult meningioma cases and 5565 population controls, suggested positive associations for ever vs. never exposure for several metals with the strongest association observed for iron exposure (OR = 1.26, 95% CI 1.0–1.58) particularly among women (OR = 1.70, 95% CI 1.0–2.89). Positive trends emerged for both cumulative exposure and duration. Associations persisted after consideration of other occupational metal co-exposures. In this article, we report on associations between glioma and occupational exposure to five metals- lead, cadmium, nickel, chromium and iron- as well as to welding fumes, using the INTEROCC data.

## Methods

### Study population

The INTEROCC study is a multicenter population-based case–control study including 10 centers from 7 countries (Australia, Canada, France, Germany, Israel, New Zealand and the United Kingdom [UK]) participating in the INTERPHONE study [23]. For most countries, eligible cases were all patients between 30 and 59 years of age and diagnosed with a primary glioma or meningioma tumor between 2000 and 2004. In Germany and the UK, the upper age limit was 69 years while Israel had no upper age limit for recruitment. Only subjects aged 30 to 69 years were included in the present analyses. The procedures for ascertainment of cases and detailed study methods have been described previously [23]. The present analysis is confined to glioma cases. Population controls were randomly selected from electoral lists (Australia, Canada-Montreal, France, New Zealand), population-based registries (Canada-Vancouver, Germany, Israel), patient lists (UK), or random digit dialing (Canada-Ottawa). They were frequency or individually matched to cases by sex, age (within 5 years) and center. In total, 2054 glioma cases and 5601 controls were recruited in the 7 countries. Participation rates were 68% for glioma cases and 50% for controls.

### Data collection

Trained interviewers conducted face-to-face interviews with study participants using a computer-assisted questionnaire. When the study subject had died or was too ill to be interviewed, the interview was conducted with a proxy respondent. Detailed information was collected on socio-economic and lifestyle characteristics (including lifetime use of mobile phones, smoking habits, and occupational history), and medical history [23]. An occupational calendar was used to collect lifetime work histories, covering all jobs held by participants for more than 6 months. It elicited information on the job title, description of tasks, and the start and end year for each job, and, in some countries, company name and description of activities of the company. Each job held by subjects was coded according to international occupation and industry classifications: the International Standard Classification of Occupations editions 1968 (ISCO68 [30]) and 1988 (ISCO88 [31]), and the International Standard Industrial Classification of All Economic Activities, revision 2 (ISIC71) [32]. Common coding guidelines were provided to each center in order to foster homogeneous coding practices. An inter-rater trial was conducted at the start of coding and results were discussed with each center in a further effort to ensure consistency of coding [33].

### Occupational exposure assessment

Occupational exposure to the agents was assessed using a modified version of the Finnish job exposure matrix FINJEM [34]. The Finnish occupation classification system contains 311 major occupational groups and covers the calendar period 1945 to 2003, divided into several sub-periods. FINJEM can translate an occupational history into a history of exposure to about 90 chemical, physical, behavioral, microbiological, ergonomic and psychosocial factors, including the 6 exposures under investigation in this paper. FINJEM provides two exposure estimates for each specific combination of occupation, calendar sub-period, and agent: the proportion of workers in that occupation who were considered to be exposed to the agent (P) and the mean level of exposure among the exposed (L) expressed in units of concentration. When P was considered to be less than 5%, the level in FINJEM was set to zero. The estimates of P and L were based on exposure measurements, hazard surveys, and the judgements by Finnish occupational hygienists.

Since FINJEM uses the Finnish occupational coding system and the INTEROCC work histories were coded according to international classifications, it was necessary to develop a “crosswalk” between the Finnish codes and the ISCO68. FINJEM was further modified for our purposes with, for example, the time window 1960–1984 split into pre and post-1974 periods. Also, specific FINJEM entries were modified for some of the exposures of interest to increase consistency and specificity of exposure assessment. For instance, FINJEM was modified by using expert-based exposure estimates developed for a population-based case–control study of lung cancer in Montréal. Assignments of exposure were peer reviewed by an international panel of occupational hygienists to ensure that the revised JEM estimates better reflected the prevailing exposure patterns in the seven participating countries. This modified instrument, referred to as the INTEROCC JEM, is described in Van Tongeren et al. [35].

### Statistical analyses

From the 2054 glioma cases and 5601 controls recruited, 1856 cases and 5189 controls met the 30–69 years age restriction. A small number of participants (56 cases, 29 controls) were excluded as they had a personal or family history of neurofibromatosis or tuberous sclerosis, or had missing or erroneous occupational history information, yielding 1800 glioma cases and 5160 controls for analysis.

For each exposure, three indices were defined for the main analysis: i) ever vs. never exposed; ii) lifetime cumulative exposure; and iii) duration of exposure. As noted above, for each job, the INTEROCC JEM provides the probability (P) that a worker in that occupation was exposed to that agent. There are several approaches for deriving an “ever exposure” variable from the INTEROCC JEM. If we designate as exposed to a given agent all those who worked in an occupation with even a small probability of exposure according to the JEM, say as low as 5%, it would be very sensitive, but most of the subjects labelled as exposed would have had a low probability of exposure. At the other extreme, if we use a high value of P as the threshold, say 95%, then it would be very specific, but a large fraction of workers truly exposed would be labelled as unexposed. The trade-off between sensitivity and specificity also has implications for the estimated prevalence of exposure. In order to give greater weight to sensitivity than specificity, but not to exaggerate this choice unduly, we used as the a priori threshold *P* ≥ 25%. Thus, ever exposure to a given agent was defined as having held at least one job with a probability of exposure of at least 25% and for at least 1 year. Subjects who had held jobs with a probability of exposure of less than 25% but greater than 5%, or for less than 1 year were considered to be of “uncertain” exposure status and were assigned to a separate category (not reported). The lifetime cumulative exposure index was defined among ever exposed (corresponding to the ever definition criteria) as the sum of the product of the probability of exposure (P), the level of exposure (L), and the duration for each job held by a subject. The continuous cumulative exposure index was categorized according to tertiles of the distribution among exposed controls. We also calculated the total duration of exposure, defined as the sum of the duration of exposure for each job held by a subject (corresponding to the ever definition criteria) minus the possible overlap period between two jobs. To allow sufficient time between occupational exposure and disease onset, all exposures that had occurred within 5 years of the reference date (age at diagnosis for cases and age at interview for controls) were not taken into account, therefore establishing a lag period of 5 years.

For all analyses, the reference category included subjects who had never been exposed to the specific chemical agent of interest. Because of the exploratory nature of these analyses, each exposure was considered independently.

Associations between glioma and each of the 6 exposures (5 metals plus welding fumes) were estimated using conditional logistic regression stratified by sex, age (5-year categories) and center. Further, all analyses were adjusted a priori for the following variables: i) age as a continuous variable (to remove any residual confounding due to age in the strata definition), ii) the maximum education level attained by the subject or her/his spouse (primary, intermediate college, tertiary), iii) the Standard International Occupational Prestige Scale (SIOPS), expressed as a time-weighted average across all jobs [36] iv) antecedents of atopy, defined as ever medical diagnosis of allergy, asthma and/or eczema (recognized as associated with glioma and exposure to metals [37]) and v) the respondent status (subject him/herself vs. proxy respondent).

Missing values for the maximum level of education attained in the household were imputed to the middle category “intermediate college” (14 subjects). Missing values for the SIOPS variable, occurring when the occupation was unknown, were imputed to the median value in the corresponding subject strata of age (5-year categories), sex, center and maximum level of education attained in the household (104 subjects). When variables constituting the “antecedents of atopy” variable were missing (22 subjects), the latter was imputed as “none”.

#### Sensitivity analyses

Sensitivity analyses were conducted to examine the influence of results using different thresholds for probability of exposure, duration of exposure, and lag period including using alternate probabilities of exposure of *P* ≥ 5% and 50%, duration of exposure of 5 years, and lag periods of 1 and 10 years. A separate set of analyses was conducted defining the reference category as never exposed to any metal nor to welding fumes. Further analyses were conducted restricted to: i) male participants; ii) female participants; iii) high-grade glioma cases; iv) glioblastoma cases; and v) participants who completed the study interview without a proxy respondent. Analyses adjusting for cigarette smoking status at diagnosis/interview (current, ex-smokers, never smokers) and marital status (married vs. others) were also conducted. Sensitivity analyses excluding one by one each of the a priori confounders from the main results model were also performed.

Ethics approval was obtained from all appropriate national and regional research ethics boards, including the Ethical Review Board of IARC (Lyon) for INTERPHONE and the Municipal Institute for Medical Investigation (IMIM) Barcelona for INTEROCC. All participants provided written informed consent.

## Results

The majority of cases (62.0%) were males whereas controls were more often females (55.3%) (Table 1). The mean (standard deviation) age of participants ranged from 50.6 (10.0) to 51.2 (9.7) years among cases and controls, respectively. Participants generally had a primary or secondary level of education, were married, and about half were never smokers. The UK and Germany contributed the majority of participants. Proxy respondents were used for 14.3% of glioma cases and 0.4% of controls.

**Table 1:** Selected characteristics of glioma cases and controls, INTEROCC study, 2000–2004*

Variable	Glioma cases	Controls
	(*n* = 1800)	(*n* = 5160)
Age, mean (SD)	50.6 (10.0)	51.2 (9.7)
Males, n (%)	1116 (62.0)	2308 (44.7)
Education, n (%)		
Primary/secondary	766 (42.6)	2253 (43.7)
Intermediate college/professional	391 (21.7)	1085 (21.0)
Tertiary	643 (35.7)	1822 (35.3)
Proxy respondent, n (%)	257 (14.3)	23 (0.4)
Country, n (%)		
Australia	277 (15.4)	665 (12.9)
Canada	169 (9.4)	649 (12.6)
France	93 (5.2)	471 (9.1)
Germany	366 (20.3)	1494 (29.0)
Israel	282 (15.7)	698 (13.5)
New Zealand	75 (4.2)	160 (3.1)
United Kingdom	538 (29.9)	1023 (19.8)
Tumor histology, n (%)		
Glioblastomas	848 (47.1)	-
Other	952 (52.9)	-
Occupational Prestige Scale (SIOPS), mean (SD)	43.2 (11.5)	44.0 (11.8)
Marital status, n (%)		
Single	159 (8.9)	431 (8.4)
Married	1422 (79.3)	4035 (78.3)
Divorced	142 (7.9)	494 (9.6)
Widowed	62 (3.5)	190 (3.7)
Don’t know	8 (0.4)	5 (0.1)
Missing	7 (−)	5 (−)
Smoking status		
Never	906 (50.3)	2521 (48.9)
Ex-smoker	386 (21.4)	1212 (23.5)
Current	508 (28.2)	1427 (27.7)
Asthma, hay fever, eczema, n (%)	416 (23.1)	1380 (26.7)

*Cases were statistically different from controls at *p* < 0.05 in terms of the following variables: age, sex, proxy respondent, country, occupational prestige, marital status, and asthma, hay fever and/or eczema

There were high percentages of overlap between exposure to each of the metals and welding fumes (Table 2). In general, cases had a higher prevalence of (ever) exposure to the various metals and welding fumes than controls (Table 3). Exposure prevalence ranged from 1.7–2.2% for cadmium up to 10.2–13.6% for iron among controls and cases, respectively.

**Table 2:** Degree of bivariate overlap in exposure to the various metals and welding fumes in INTEROCC, in glioma cases and controls combined, 2000-2004^a^

	Cadmium	Chromium	Iron	Lead	Nickel	Welding fumes
*n*	*130*	*537*	*772*	*546*	*659*	*570*
Cadmium	-	7.1	6.6	10.4	7.4	2.6
Chromium	29.2	-	69.6	40.3	79.8	66.0
Iron	39.2	100	-	64.1	95.6	100
Lead	43.8	41.0	45.3	-	44.5	53.0
Nickel	37.7	98.0	81.6	53.7	-	82.8
Welding fumes	11.5	70.0	73.8	55.3	71.6	-

^a^Values of cells in each column represent the proportion of participants with exposure to the agent in the column heading who were also exposed to the agent in the corresponding row. For welding fumes, exposures to other metals could come from welding activities, from other activities in the same job and/or from activities in other jobs

**Table 3:** Adjusted^a^ odds ratios (OR) and 95% confidence intervals (CI) for the risk of glioma and exposure to each of the 5 metals and welding fumes for a duration ≥1 year, a lag of 5 years and *P* ≥ 25%

Exposure	Glioma cases	Controls	OR (95% CI)
	n (%)	n (%)	
Cadmium			
Non-exposed	1741 (96.7)	5039 (97.7)	1.0 (Ref)
Cumulative exposure	40 (2.2)	90 (1.7)	1.1 (0.7–1.6)
≤ 111.4 μg/m^3^	12 (0.7)	30 (0.6)	1.0 (0.5–1.9)
> 111.4 to ≤343.8 μg/m^3^	19 (1.1)	31 (0.6)	1.6 (0.9–2.8)
> 343.8 μg/m^3^	9 (0.5)	29 (0.6)	0.7 (0.3–1.5)
Duration of exposure			
1–4 years	20 (1.1)	44 (0.9)	1.1 (0.6–1.8)
5–9 years	10 (0.6)	19 (0.4)	1.4 (0.6–3.3)
≥ 10 years	10 (0.6)	27 (0.5)	0.8 (0.4–1.8)
Restricted to males	31 (2.8)	56 (2.4)	1.1 (0.7–1.8)
High grade cases	25 (2.0)	90 (1.7)	0.9 (0.6–1.5)
Glioblastomas	18 (2.1)	90 (1.7)	0.9 (0.5–1.5)
Self-respondents	38 (2.5)	89 (1.7)	1.1 (0.7–1.7)
Chromium			
Non-exposed	1508 (83.8)	4535 (87.9)	1.0 (Ref)
Cumulative exposure	178 (9.9)	359 (7.0)	0.9 (0.7–1.1)
≤ 445.5 μg/m^3^	61 (3.4)	121 (2.3)	0.9 (0.6–1.3)
> 445.5 to ≤3000 μg/m^3^	57 (3.2)	119 (2.3)	0.9 (0.6–1.3)
> 3000 μg/m^3^	60 (3.3)	119 (2.3)	0.9 (0.6–1.3)
Duration of exposure			
1–4 years	41 (2.3)	95 (1.8)	0.9 (0.6–1.3)
5–9 years	36 (2.0)	81 (1.6)	0.8 (0.5–1.2)
≥ 10 years	101 (5.6)	183 (3.5)	1.0 (0.8–1.4)
Restricted to males	175 (15.7)	337 (14.6)	0.9 (0.7–1.2)
High grade cases	124 (9.9)	359 (7.0)	0.9 (0.7–1.2)
Glioblastomas	83 (9.8)	359 (7.0)	0.8 (0.6–1.1)
Self-respondents	150 (9.7)	358 (7.0)	0.9 (0.7–1.1)
Iron			
Non-exposed	1546 (85.9)	4604 (89.2)	1.0 (Ref)
Cumulative exposure	244 (13.6)	528 (10.2)	0.9 (0.7–1.1)
≤ 70 mg/m^3^	64 (3.6)	181 (3.5)	0.7 (0.5–1.0)
> 70 to ≤254.3 mg/m^3^	81 (4.5)	171 (3.3)	0.9 (0.7–1.2)
> 254.3 mg/m^3^	99 (5.5)	176 (3.4)	1.1 (0.8–1.5)
Duration of exposure			
1–4 years	52 (2.9)	139 (2.7)	0.8 (0.6–1.2)
5–9 years	57 (3.2)	116 (2.2)	0.9 (0.6–1.3)
≥ 10 years	135 (7.5)	273 (5.3)	0.9 (0.7–1.2)
Restricted to males	237 (21.2)	493 (21.4)	0.9 (0.7–1.1)
High grade cases	181 (14.5)	528 (10.2)	1.0 (0.8–1.2)
Glioblastomas	125 (14.7)	528 (10.2)	1.2 (0.4–3.2)
Self-respondents	211 (13.7)	527 (10.3)	0.9 (0.7–1.1)
Lead			
Non-exposed	1419 (78.8)	4296 (83.3)	1.0 (Ref)
Cumulative exposure	159 (8.8)	387 (7.5)	0.8 (0.7–1.0)
≤ 128.8 μmol/l (in blood)	45 (2.5)	128 (2.5)	0.8 (0.6–1.2)
> 128.8 to ≤413.2 μmol/l (in blood)	47 (2.6)	131 (2.5)	0.7 (0.5–1.0)
> 413.2 μmol/l (in blood)	67 (3.7)	128 (2.5)	1.0 (0.7–1.3)
Duration of exposure			
1–4 years	58 (3.2)	140 (2.7)	1.0 (0.7–1.4)
5–9 years	32 (1.8)	81 (1.6)	0.7 (0.4–1.1)
≥ 10 years	69 (3.8)	166 (3.2)	0.8 (0.6–1.1)
Restricted to males	151 (13.5)	321 (13.9)	0.9 (0.7–1.1)
High grade cases	121 (9.7)	387 (7.5)	0.9 (0.7–1.2)
Glioblastomas	85 (10.0)	387 (7.5)	0.9 (0.7–1.2)
Self-respondents	135 (8.7)	387 (7.5)	0.8 (0.7–1.0)
Nickel			
Non-exposed	1534 (85.2)	4585 (88.9)	1.0 (Ref)
Cumulative exposure	215 (11.9)	444 (8.6)	0.9 (0.8–1.1)
≤ 317.2 μg/m^3^	55 (3.1)	149 (2.9)	0.8 (0.5–1.1)
> 317.2 to ≤951.3 μg/m^3^	72 (4.0)	148 (2.9)	0.9 (0.7–1.3)
> 951.3 μg/m^3^	88 (4.9)	147 (2.8)	1.1 (0.8–1.5)
Duration of exposure			
1–4 years	46 (2.6)	128 (2.5)	0.8 (0.5–1.1)
5–9 years	51 (2.8)	104 (2.0)	0.9 (0.6–1.3)
≥ 10 years	118 (6.6)	212 (4.1)	1.0 (0.8–1.3)
Restricted to males	209 (18.7)	410 (17.8)	0.9 (0.8–1.2)
High grade cases	155 (12.4)	444 (8.6)	1.0 (0.8–1.2)
Glioblastomas	107 (12.6)	444 (8.6)	0.9 (0.7–1.2)
Self-respondents	184 (11.9)	443 (8.6)	0.9 (0.8–1.1)
Welding fumes			
Non-exposed	1546 (85.9)	4606 (89.3)	1.0 (Ref)
Cumulative exposure	182 (10.1)	388 (7.5)	0.9 (0.7–1.1)
≤ 180 mg/m^3^	63 (3.5)	136 (2.6)	0.9 (0.6–1.2)
> 180 to ≤684 mg/m^3^	54 (3.0)	123 (2.4)	0.8 (0.6–1.2)
> 684 mg/m^3^	65 (3.6)	129 (2.5)	1.0 (0.7–1.4)
Duration of exposure			
1–4 years	44 (2.4)	110 (2.1)	0.8 (0.6–1.2)
5–9 years	39 (2.2)	77 (1.5)	0.9 (0.6–1.4)
≥ 10 years	99 (5.5)	201 (3.9)	0.9 (0.7–1.2)
Restricted to males	178 (15.9)	375 (16.2)	0.9 (0.7–1.1)
High grade cases	131 (10.5)	388 (7.5)	0.9 (0.7–1.2)
Glioblastomas	95 (11.2)	388 (7.5)	0.9 (0.7–1.2)
Self-respondents	157 (10.2)	388 (7.6)	0.9 (0.7–1.1)

^a^Adjusted for age, educational level, occupational prestige, atopy, and respondent status

Associations between exposure to each of the 5 metals and welding fumes, and glioma risk are also presented in Table 3. Once covariates were included in the models, there was no association between ever exposure to any of the agents and glioma risk, with ORs ranging from 0.8 (95% CI 0.7–1.0) for lead to 1.1 (95% CI 0.7–1.6) for cadmium. There was also no evidence for a trend according to categories of cumulative exposure, including in the highest tertile, where ORs were generally near 1.0. Analyses based on three categories of duration of exposure (1–4, 5–9, ≥10 years) yielded similar findings. The only elevated, but non-significant ORs were observed for cadmium, in the second tertile of cumulative exposure (OR = 1.6, 95% CI 0.9–2.8) and among subjects exposed 5–9 years (OR = 1.4, 95%CI 0.6–3.3). Some ORs in low exposure categories were non-significantly decreased, as for lead and iron.

Results were similar for all of the agents upon restriction of the analysis to males or to females (results not shown), high-grade glioma cases, glioblastoma cases, or to self-respondents (Table 3). There was also no evidence for an association according to alternate definitions of P, duration or lag time for any of the agents (Additional file 1: Table S1). This held true when using an unexposed category that excluded exposure to any of the metals under study. Excluding one by one each of the a priori confounders from the main model had a marginal impact on findings. There was no effect modification by education or smoking history (not shown).

Upon further investigation of cadmium according to different durations and probabilities of exposure, and lag times (Additional file 1: Table S1), a significant increase in risk was observed in the first tertile of exposure for a lag of 5 years, a duration ≥5 years and a probability ≥5%. Some ORs were greater than one following the various exposure scenarios but no clear pattern emerged and numbers were small.

## Discussion

We investigated the relationship between glioma and occupational exposure to five metals- lead, cadmium, nickel, chromium and iron- as well as to welding fumes, in the seven-country INTEROCC study. Overall, there was no evidence of an association between ever exposure to any of the metals or welding fumes and glioma, with ORs generally near 1.0. No associations were observed according to categories of cumulative exposure or duration of exposure or to a range of alternate assumptions regarding exposure modeling or lag time. Results were consistent across an array of sensitivity analyses.

In the IARC Monograph program on the evaluation of carcinogenic risks to humans, cadmium and cadmium compounds, chromium-6 compounds, nickel compounds, welding fumes, and occupational exposure during iron and steel founding have been classified as human carcinogens (Group 1) while inorganic lead is currently classified as a probably carcinogenic (Group 2A) [38–41]. However, for none of these has the brain been identified as a target site.

Previous results from epidemiological studies examining associations between occupational metal exposure and brain tumors are mixed. An elevated OR was observed among men, but not women, with occupational activities involving metals in a 6-country study of glioma risk (OR = 1.24, 95% CI 0.96–1.62) (*n* = 1178 cases, 1987 controls), but no association emerged when self-reports of exposure to metal or metal compounds were used [17]. An analysis based on data from the German INTERPHONE study group, which are subsumed in the present analysis, suggested no association between having ever worked in an occupational sector involving metal production and glioma risk or according to duration of work in the sector [18]. One U.S. study including 489 glioma cases and 799 controls reported no overall association between occupational lead exposure as assessed using a quantitative lead exposure database and detailed job-level information [19], although there was some evidence of modification of the association with glioblastoma multiforme by single nucleotide polymorphisms in genes related with oxidative stress [20]. A positive association was observed between occupational lead exposure and brain cancer mortality in the National Longitudinal Mortality Study (OR for any exposure = 1.56, 95% CI 1.00–2.43, based on 119 brain cancer deaths) [11]. In a study linking JEM-based exposure to individual metals and the Swedish national cancer register [10], no clear association emerged between occupational chromium/nickel, lead, or metallic compound exposure and glioma risk among 2.8 million men employed in 1970 (*n* = 3363 glioma cases). In contrast, in another study [14], some elevated standardized incidence ratios (SIRs) for brain cancer cancer (*n* = 693) were observed in a cohort of 413,000 Finnish women with blue collar occupations in 1970 entailing exposure to iron (SIR = 2.15, 95% CI 0.96–4.80), chromium compounds (SIR = 1.51, 95% CI 0.85–2.67), lead (SIR = 1.27, 95% CI 0.81–2.01), and cadmium (SIR = 1.24, 95% CI 0.72–2.22). There was an elevated risk of brain cancer mortality (standardized mortality ratio = 9.14, 95% CI 1.81–22.09) in a cohort of 1193 male chromium platers in Japan, although this results was based on only three brain cancer deaths [15]. No association was found with brain cancer mortality in a cohort of 9122 workers with measured blood lead levels in Great Britain, based on 23 brain cancer deaths [42]. There was a positive, though imprecise association between ever occupational exposure to lead dust and fumes, assigned based on a combination of expert intensity ratings and inspection measurements, in a Shanghai general population cohort (RR = 1.8, 95% CI 0.7–4.8, *n* = 77 incident brain cancer cases).

The cytotoxicity of cadmium has been demonstrated [43]. It can alter the brain function and has been linked to cancer development in experimental animals. Studies on humans are suggestive of associations with cancer sites such as the prostate and the kidney [44], but little evidence has accrued with respect to brain cancer. In our study, cadmium was the only agent showing some risk estimates departing from the null value. However, no clear patterns of risk emerged and this may result from low numbers and statistical instability.

Most studies reporting on welding found no association with brain cancer [45–48]. A suggestive association emerged in a Canadian study of 1009 incident cases of brain cancer and 5039 controls for welding over 20 years or more [49].

Strengths of the current study include the large sample size with 1800 glioma cases and 5160 controls, detailed data on participant lifetime occupational history, objectively assigned exposure estimates, information on potential confounders, as well as information on tumor histology and grade. Nevertheless, we found no evidence of an association between metals or welding fumes, and glioma risk. The null associations observed may reflect various features of the study, including low prevalence and misclassification of exposure. However, associations have been previously been observed in INTEROCC, including between occupational exposure to metal, particularly iron, as well as to oil mist, and risk of meningioma [27, 29]. In contrast with the latter positive findings for meningioma, there were no associations observed for glioma risk here or in other analyses assessing occupational exposure to solvents, combustion products, dusts and other chemical agents overall or according to sex, tumor histology, tumor grade, respondent status, and considering various exposure modeling approaches or lag time [24, 25].

JEMs entail assigning the same set of exposures to all subjects holding a given occupational title. They thus overlook idiosyncratic jobs circumstances that can influence exposure levels. This typically leads to non-differential misclassification of exposure, diminishing the opportunity to detect a true association [50]. In a case–control study of occupational exposure to lead there was moderate agreement in exposure estimates between an expert assessment of detailed work history versus a JEM approach, with the JEM demonstrating higher specificity (~0.9) than sensitivity (~0.5) [51]. Several steps were undertaken in the study to adapt FINJEM for application to the INTEROCC study, including peer review by an international panel of occupational hygienists to ensure that INTEROCC JEM estimates better reflected the prevailing exposure patterns in the seven participating countries [35]. A comprehensive comparison of FINJEM exposure estimates to those potentially encountered in other countries could not be carried out. However, assessments were found to be reasonably comparable to those in a large population-based case–control study in Montreal, especially for metals and welding fumes [52].

Recall bias is always a concern in interview-based studies, but as in this case exposure relied on reporting of the occupational history, similar degrees of accuracy are assumed between cases and controls. There was a relatively high proportion of proxy interviews in the case group, but findings were similar in analyses including and excluding proxy respondents.

As a whole, our study covers ages at diagnosis from 30 to 69 years, with some countries limited to age 59. Based on the SEER registry, the median age at diagnosis of glioma (all subtypes combined) is 59 years. For all subtypes other than glioblastoma and gliosarcoma, the median age is below that [53]. It is possible that the low numbers for older age groups in our study limited our ability to detect associations for exposures longer than covered here and for subtypes than tend to develop at more advanced ages.

## Conclusions

Findings from this large population-based study using a detailed exposure assessment provide no evidence of an association between lifetime occupational exposure to any of the five metals under study, or to welding fumes, and risk of developing glioma.

## Additional file

Additional file 1: Table S1. Adjusted^a^ odds ratios (OR) and 95% confidence intervals (CI) for the risk of glioma and exposure to each of the 5 metals and welding fumes, according to different durations and probabilities of exposure, and lag times. (DOCX 27 kb)

## Abbreviations

CI: Confidence interval
ELF: Extremely low frequency magnetic fields
ISCO: International standard classification of occupations
ISIC: International standard industrial classification
JEM: Job exposure matrix
L: Level of exposure
OR: Odds ratio
P: Probability of exposure
RF-EMF: Radiofrequency electromagnetic fields
RR: Relative risk
SIOPS: Standard international occupational prestige scale
SIR: Standardized incidence ratio
UK: United Kingdom
