# Supplementary material for: Lifetime occupational exposure to metals and welding fumes, and risk of glioma: a 7-country population-based case–control study
Source: Environ Health. 2017 Aug 25;16:90. doi: 10.1186/s12940-017-0300-y (PMC5574088; doi:10.1186/s12940-017-0300-y)
Supplement: Additional file 1: Table S1. — Adjusteda odds ratios (OR) and 95% confidence intervals (CI) for the risk of glioma and exposure to each of the 5 metals and welding fumes, according to different durations and probabilities of exposure, and lag times. (DOCX 27 kb) [file 12940_2017_300_MOESM1_ESM.docx]

**Additional file 1:** **Table S1.** Adjusted^a^ odds ratios (OR) and 95% confidence intervals (CI) for the risk of glioma and exposure to each of the 5 metals and welding fumes, according to different durations and probabilities of exposure, and lag times

|  | Cadmium | | | Chromium | | | Iron | | | Lead | | | Nickel | | | Welding fumes | | |
| --- | --- | --- | --- | --- | --- | --- | --- | --- | --- | --- | --- | --- | --- | --- | --- | --- | --- | --- |
|  | **nCa** | **nCo** | **OR (95%CI)** | **nCa** | **nCo** | **OR (95%CI)** | **nCa** | **nCo** | **OR (95%CI)** | **nCa** | **nCo** | **OR (95%CI)** | **nCa** | **nCo** | **OR (95%CI)** | **nCa** | **nCo** | **OR (95%CI)** |
| Lag of 1 yr Duration ≥ 1yr P≥25% |  |  |  |  |  |  |  |  |  |  |  |  |  |  |  |  |  |  |
| Non-exposed | 1741 | 5039 |  | 1508 | 4535 |  | 1546 | 4604 |  | 1419 | 4296 |  | 1534 | 4585 |  | 1546 | 4606 |  |
| Exposed | 40 | 90 | 1.1(0.7-1.6) | 180 | 362 | 0.9(0.7-1.1) | 247 | 531 | 0.9(0.8-1.1) | 159 | 390 | 0.8(0.7-1.0) | 217 | 448 | 0.9(0.8-1.1) | 183 | 391 | 0.9(0.7-1.1) |
| Tertile 1 | 13 | 30 | 1.1(0.5-2.1) | 60 | 121 | 0.9(0.6-1.3) | 67 | 178 | 0.8(0.6-1.1) | 45 | 129 | 0.8(0.6-1.2) | 54 | 150 | 0.7(0.5-1.0) | 60 | 131 | 0.9(0.6-1.2) |
| Tertile 2 | 18 | 30 | 1.5(0.8-2.7) | 58 | 121 | 0.9(0.7-1.3) | 76 | 177 | 0.8(0.6-1.1) | 46 | 132 | 0.7(0.5-1.0) | 75 | 149 | 1.0(0.7-1.3) | 62 | 131 | 0.9(0.6-1.2) |
| Tertile 3 | 9 | 30 | 0.6(0.3-1.5) | 62 | 120 | 0.9(0.7-1.3) | 104 | 176 | 1.2(0.9-1.5) | 68 | 129 | 1.0(0.7-1.4) | 88 | 149 | 1.1(0.8-1.5) | 61 | 129 | 1(0.7-1.4) |
|  |  |  |  |  |  |  |  |  |  |  |  |  |  |  |  |  |  |  |
| Lag of 5 yrs Duration ≥ 1yr  P≥5% |  |  |  |  |  |  |  |  |  |  |  |  |  |  |  |  |  |  |
| Non-exposed | 1741 | 5039 |  | 1508 | 4535 |  | 1546 | 4604 |  | 1419 | 4296 |  | 1534 | 4585 |  | 1546 | 4606 |  |
| Exposed | 54 | 113 | 1.1(0.8-1.6) | 286 | 613 | 0.9(0.8-1.1) | 248 | 543 | 0.9(0.7-1.1) | 372 | 831 | 0.9(0.8-1.1) | 260 | 560 | 0.9(0.8-1.1) | 248 | 541 | 0.9(0.7-1.1) |
| Tertile 1 | 19 | 38 | 1.2(0.6-2.1) | 85 | 205 | 0.9(0.7-1.2) | 63 | 180 | 0.7(0.5-1.0) | 115 | 276 | 0.9(0.7-1.2) | 69 | 189 | 0.8(0.6-1.0) | 77 | 182 | 0.9(0.6-1.1) |
| Tertile 2 | 16 | 37 | 1.0(0.6-1.9) | 95 | 204 | 0.9(0.6-1.1) | 79 | 183 | 0.8(0.6-1.1) | 129 | 279 | 1.0(0.8-1.3) | 87 | 185 | 0.9(0.7-1.2) | 88 | 179 | 0.9(0.7-1.3) |
| Tertile 3 | 19 | 38 | 1.2(0.6-2.1) | 106 | 204 | 1.0(0.7-1.3) | 106 | 180 | 1.2(0.9-1.5) | 128 | 276 | 0.9(0.7-1.1) | 104 | 186 | 1.1(0.8-1.4) | 83 | 180 | 0.9(0.7-1.2) |
|  |  |  |  |  |  |  |  |  |  |  |  |  |  |  |  |  |  |  |
| Lag of 5 yrs Duration ≥ 5yrs  P≥5% |  |  |  |  |  |  |  |  |  |  |  |  |  |  |  |  |  |  |
| Non-exposed | 1741 | 5039 |  | 1508 | 4535 |  | 1546 | 4604 |  | 1419 | 4296 |  | 1534 | 4585 |  | 1546 | 4606 |  |
| Exposed | 31 | 55 | 1.4(0.9-2.2) | 220 | 453 | 0.9(0.7-1.1) | 195 | 395 | 0.9(0.8-1.2) | 268 | 571 | 0.9(0.8-1.1) | 205 | 408 | 1.0(0.8-1.2) | 195 | 393 | 0.9(0.8-1.2) |
| Tertile 1 | 15 | 19 | 2.2(1.1-4.6) | 69 | 151 | 0.9(0.6-1.2) | 50 | 132 | 0.7(0.5-1.0) | 92 | 190 | 1.0(0.7-1.3) | 59 | 135 | 0.8(0.6-1.2) | 61 | 131 | 0.8(0.6-1.2) |
| Tertile 2 | 7 | 18 | 0.7(0.3-1.9) | 70 | 152 | 0.9(0.6-1.2) | 73 | 132 | 1.1(0.8-1.5) | 83 | 191 | 0.9(0.7-1.2) | 78 | 138 | 1.1(0.8-1.5) | 68 | 132 | 1.0(0.7-1.4) |
| Tertile 3 | 9 | 18 | 1.3(0.5-3.0) | 81 | 150 | 1.0(0.7-1.3) | 72 | 131 | 1.1(0.8-1.5) | 93 | 190 | 0.9(0.7-1.2) | 68 | 135 | 1.0(0.7-1.3) | 66 | 130 | 1.0(0.7-1.4) |
|  |  |  |  |  |  |  |  |  |  |  |  |  |  |  |  |  |  |  |
| Lag of 5 yrs Duration ≥ 5yrs P≥25% |  |  |  |  |  |  |  |  |  |  |  |  |  |  |  |  |  |  |
| Non-exposed | 1741 | 5039 |  | 1508 | 4535 |  | 1546 | 4604 |  | 1419 | 4296 |  | 1534 | 4585 |  | 1546 | 4606 |  |
| Exposed | 20 | 46 | 1.1(0.6-1.9) | 137 | 264 | 0.9(0.7-1.2) | 192 | 389 | 0.9(0.8-1.1) | 101 | 247 | 0.7(0.6-1.0) | 169 | 316 | 1.0(0.8-1.2) | 138 | 278 | 0.9(0.7-1.2) |
| Tertile 1 | 10 | 16 | 1.9(0.8-4.5) | 40 | 88 | 0.8(0.5-1.2) | 47 | 130 | 0.6(0.4-0.9) | 24 | 84 | 0.5(0.3-0.9) | 54 | 106 | 0.9(0.6-1.3) | 45 | 93 | 0.8(0.6-1.2) |
| Tertile 2 | 2 | 15 | 0.2(0.0-1.1) | 46 | 89 | 1.0(0.7-1.5) | 73 | 129 | 1.1(0.8-1.5) | 36 | 80 | 0.9(0.6-1.4) | 56 | 105 | 1.0(0.7-1.4) | 45 | 93 | 0.9(0.6-1.4) |
| Tertile 3 | 8 | 15 | 1.3(0.5-3.4) | 51 | 87 | 1.1(0.7-1.6) | 72 | 130 | 1.1(0.8-1.5) | 41 | 83 | 0.8(0.6-1.3) | 59 | 105 | 1.1(0.8-1.6) | 48 | 92 | 1.1(0.7-1.6) |
|  |  |  |  |  |  |  |  |  |  |  |  |  |  |  |  |  |  |  |
| Lag of 5 yrs Duration ≥ 1yr  P≥50% |  |  |  |  |  |  |  |  |  |  |  |  |  |  |  |  |  |  |
| Non-exposed | 1741 | 5039 |  | 1508 | 4535 |  | 1546 | 4604 |  | 1419 | 4296 |  | 1534 | 4585 |  | 1546 | 4606 |  |
| Exposed | 17 | 51 | 0.9(0.5-1.6) | 41 | 83 | 0.9(0.6-1.3) | 238 | 512 | 0.9(0.7-1.1) | 130 | 337 | 0.8(0.6-1.0) | 2 | 2 | 1.0(0.1-9.9) | 69 | 152 | 0.9(0.6-1.2) |
| Tertile 1 | 3 | 17 | 0.4(0.1-1.6) | 11 | 32 | 0.5(0.2-1.1) | 60 | 173 | 0.7(0.5-1.0) | 32 | 113 | 0.7(0.4-1.0) | - | - | - | 21 | 50 | 0.8(0.4-1.3) |
| Tertile 2 | 8 | 18 | 1.4(0.6-3.2) | 11 | 25 | 0.8(0.4-1.6) | 79 | 169 | 0.9(0.6-1.2) | 42 | 113 | 0.8(0.5-1.1) | 1 | 2 | 0.2(0.0-6.9) | 18 | 52 | 0.7(0.4-1.2) |
| Tertile 3 | 6 | 16 | 0.9(0.3-2.4) | 19 | 26 | 1.5(0.8-3.0) | 99 | 170 | 1.1(0.9-1.5) | 56 | 111 | 0.9(0.6-1.3) | 1 | 0 | 38597(0.0-∞) | 30 | 50 | 1.2(0.7-2.0) |
|  |  |  |  |  |  |  |  |  |  |  |  |  |  |  |  |  |  |  |
| Lag of 5 yrs Duration ≥ 5yrs P≥50% |  |  |  |  |  |  |  |  |  |  |  |  |  |  |  |  |  |  |
| Non-exposed | 1741 | 5039 |  | 1508 | 4535 |  | 1546 | 4604 |  | 1419 | 4296 |  | 1534 | 4585 |  | 1546 | 4606 |  |
| Exposed | 11 | 25 | 1.3(0.6-2.8) | 30 | 56 | 1.0(0.6-1.6) | 189 | 379 | 0.9(0.8-1.2) | 88 | 207 | 0.8(0.6-1.1) | 1 | 1 | 1.7(0.1-27.7) | 44 | 101 | 0.9(0.6-1.3) |
| Tertile 1 | 5 | 9 | 2.4(0.8-7.3) | 6 | 20 | 0.5(0.2-1.4) | 44 | 127 | 0.6(0.4-0.9) | 24 | 69 | 0.7(0.4-1.1) | 0 | 1 | 0(0.0-∞) | 10 | 34 | 0.7(0.3-1.3) |
| Tertile 2 | 1 | 8 | 0.3(0.0-2.2) | 13 | 18 | 1.5(0.7-3.1) | 73 | 126 | 1.1(0.8-1.5) | 28 | 70 | 0.9(0.5-1.4) | - | - | - | 11 | 34 | 0.7(0.3-1.5) |
| Tertile 3 | 5 | 8 | 1.7(0.5-5.8) | 11 | 18 | 1.2(0.5-2.7) | 72 | 126 | 1.1(0.8-1.6) | 36 | 68 | 0.9(0.6-1.4) | 1 | 0 | 104820(0.0-∞) | 23 | 33 | 1.3(0.7-2.3) |
|  |  |  |  |  |  |  |  |  |  |  |  |  |  |  |  |  |  |  |
| Lag of 10 yrs Duration ≥ 1yr P≥25% |  |  |  |  |  |  |  |  |  |  |  |  |  |  |  |  |  |  |
| Non-exposed | 1745 | 5048 |  | 1515 | 4553 |  | 1554 | 4623 |  | 1434 | 4335 |  | 1543 | 4605 |  | 1554 | 4624 |  |
| Exposed | 38 | 83 | 1.1(0.7-1.7) | 170 | 340 | 0.9(0.7-1.2) | 237 | 509 | 0.9(0.8-1.1) | 151 | 375 | 0.8(0.7-1.0) | 206 | 426 | 0.9(0.8-1.1) | 176 | 376 | 0.9(0.7-1.1) |
| Tertile 1 | 12 | 28 | 1.0(0.5-2.0) | 58 | 115 | 0.9(0.6-1.3) | 64 | 172 | 0.7(0.5-1.0) | 42 | 127 | 0.8(0.5-1.1) | 57 | 142 | 0.8(0.5-1.1) | 62 | 128 | 0.9(0.6-1.3) |
| Tertile 2 | 16 | 28 | 1.4(0.7-2.7) | 49 | 112 | 0.8(0.6-1.2) | 78 | 167 | 0.9(0.7-1.2) | 43 | 123 | 0.7(0.5-1.0) | 67 | 143 | 1.0(0.7-1.3) | 53 | 123 | 0.8(0.6-1.2) |
| Tertile 3 | 10 | 27 | 0.8(0.4-1.9) | 63 | 113 | 1.0(0.7-1.5) | 95 | 170 | 1.1(0.8-1.5) | 66 | 125 | 1.0(0.7-1.4) | 82 | 141 | 1.1(0.8-1.5) | 61 | 125 | 1.0(0.7-1.4) |

^a^Adjusted for age, educational level, occupational prestige, antecedents of atopy, and respondent status.
